# Rare but Fatal Case of Cavitary Pneumonia Caused by Alcaligenes Faecalis

**DOI:** 10.7759/cureus.8934

**Published:** 2020-06-30

**Authors:** Rana Al-Zakhari, Maham Suhail, Basma Ataallah, Safa Aljammali, Angela Grigos

**Affiliations:** 1 Internal Medicine, Richmond University Medical Center, Staten Island, USA; 2 Internal Medicine, Zucker School of Medicine at Mather, Port Jefferson, USA; 3 Internal Medicine, Northwell Health Mather Hospital, Port Jefferson, USA

**Keywords:** alcaligenes faecalis, pathogen, fatal, pan-drug resistance

## Abstract

Alcaligenes faecalis is a gram-negative bacterium that is commonly found in the environment. This pathogen is usually transmitted in the form of droplets through ventilation equipment and nebulizers, but transmission through direct contact has also been documented in few case reports. This pathogen can cause rare but fatal infections including appendicitis, abscesses, meningitis, bloodstream infection, endocarditis, and post-operative endophthalmitis. Pan drug resistance to all commercially available antibiotics has been emerging globally. We present the case of a 66-year-old male who had respiratory failure along with multiple comorbidities. A large cavitary lesion caused by pan drug-resistant Alcaligenes faecalis was found on chest imaging. Despite the treatment with broad-spectrum antibiotics, the clinical outcome was very poor.

## Introduction

A rod-shaped and gram-negative bacterium that is commonly found in the environment has a species named Alcaligenes faecalis. It has the ability to produce an alkaline reaction in certain medium. This bacterium is oxidase-positive and also nonfermentive aerobic [1,2].

This bacterium is commonly transmitted in the form of droplets through ventilation equipment and nebulizers. Direct contact also causes an infection according to a few case reports [3,4]. Even though an infection from this opportunistic pathogen is very rare, it is still fatal [5]. Till date, pan drug resistance has been observed in A. faecalis species [6].

## Case presentation

A 66-year-old male was brought to the emergency room from the nursing home. He was found desaturating on mechanical ventilation. The patient had a medical history of chronic obstructive pulmonary disease, atrial fibrillation, morbid obesity, ventilator-dependent respiratory failure, congestive heart failure, and anoxic brain injury. The patient went into cardiac arrest, as reported by the emergency medical services, and it took 16 minutes for him to return to spontaneous circulation. The patient’s vital signs were recorded as blood pressure of 100/60 mm Hg, respiratory rate of 20 breaths per minute, temperature of 98.0 F, 100% oxygen saturation on FiO_2_ 100%, and a pulse rate of 70 beats per minute. The patient was responding to painful stimuli only minimally upon physical examination and was dependant on the ventilator. Decreased breathing sounds were revealed on the chest examination with crackles on the right side, normal S1 and S2, no murmur or gallops were heard, the abdomen was soft, no tenderness or organomegaly was noted, jugular venous pressure was not raised, bilateral lower extremities edema was seen in extremities, and normal tympanic sound was observed. Investigations were carried out in the laboratory, which showed 6.7 g/dL of hemoglobin, 6.6 mmol/L of potassium, 12.8 K/Ul of white cell count, 168 k/uL of platelets, 3.1 mg/dL of creatinine, 5.7 mmol/L of lactic acid, and 112 mg/dL of BUN (blood urea nitrogen).

**Figure 1:**
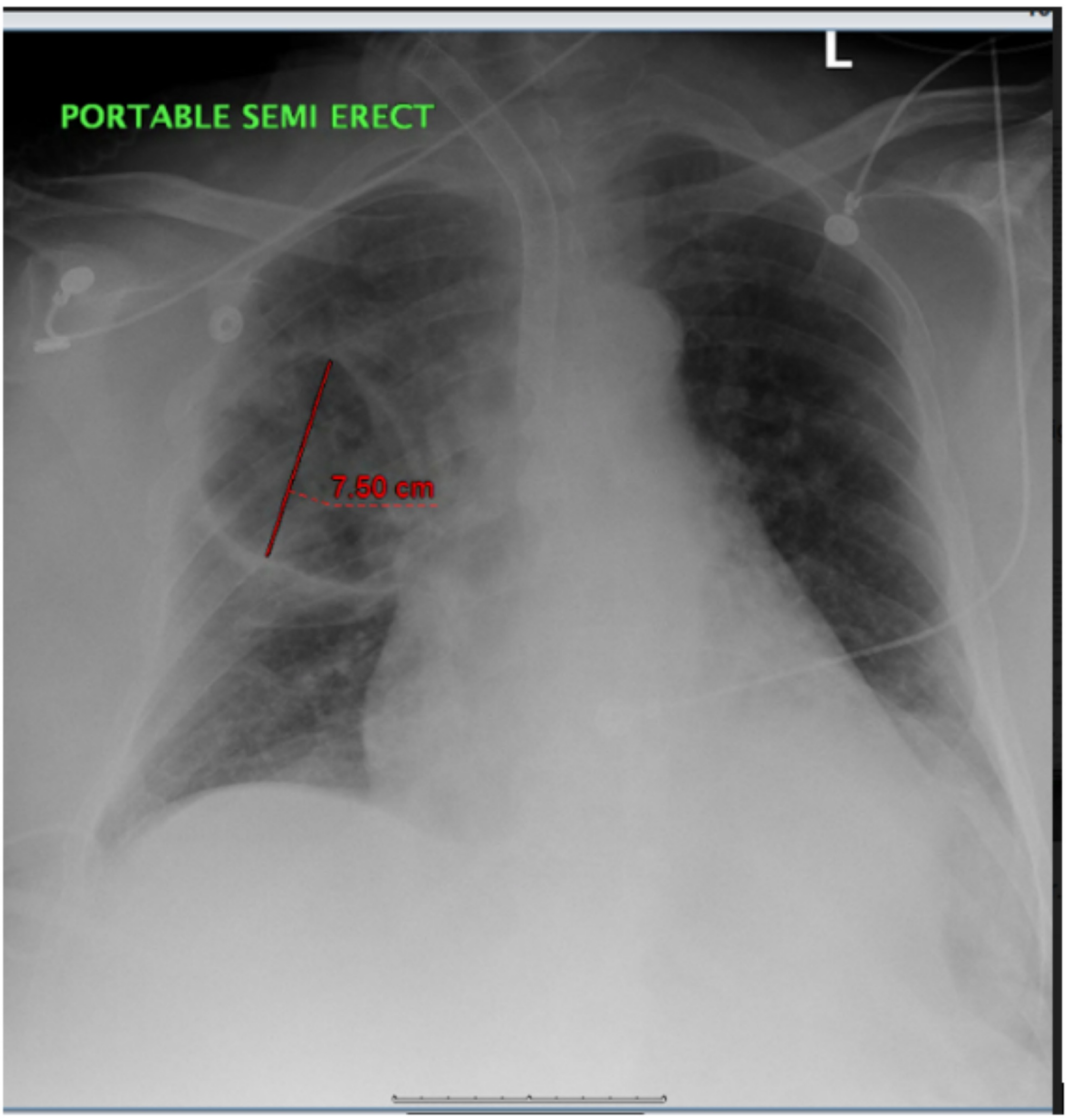
Chest X-ray: There is patchy infiltrate versus atelectasis at the left lung base. There are patchy infiltrates in the right upper lobe. There is also new thick-walled cystic airspace in the right upper lobe since the previous study.

**Figure 2:**
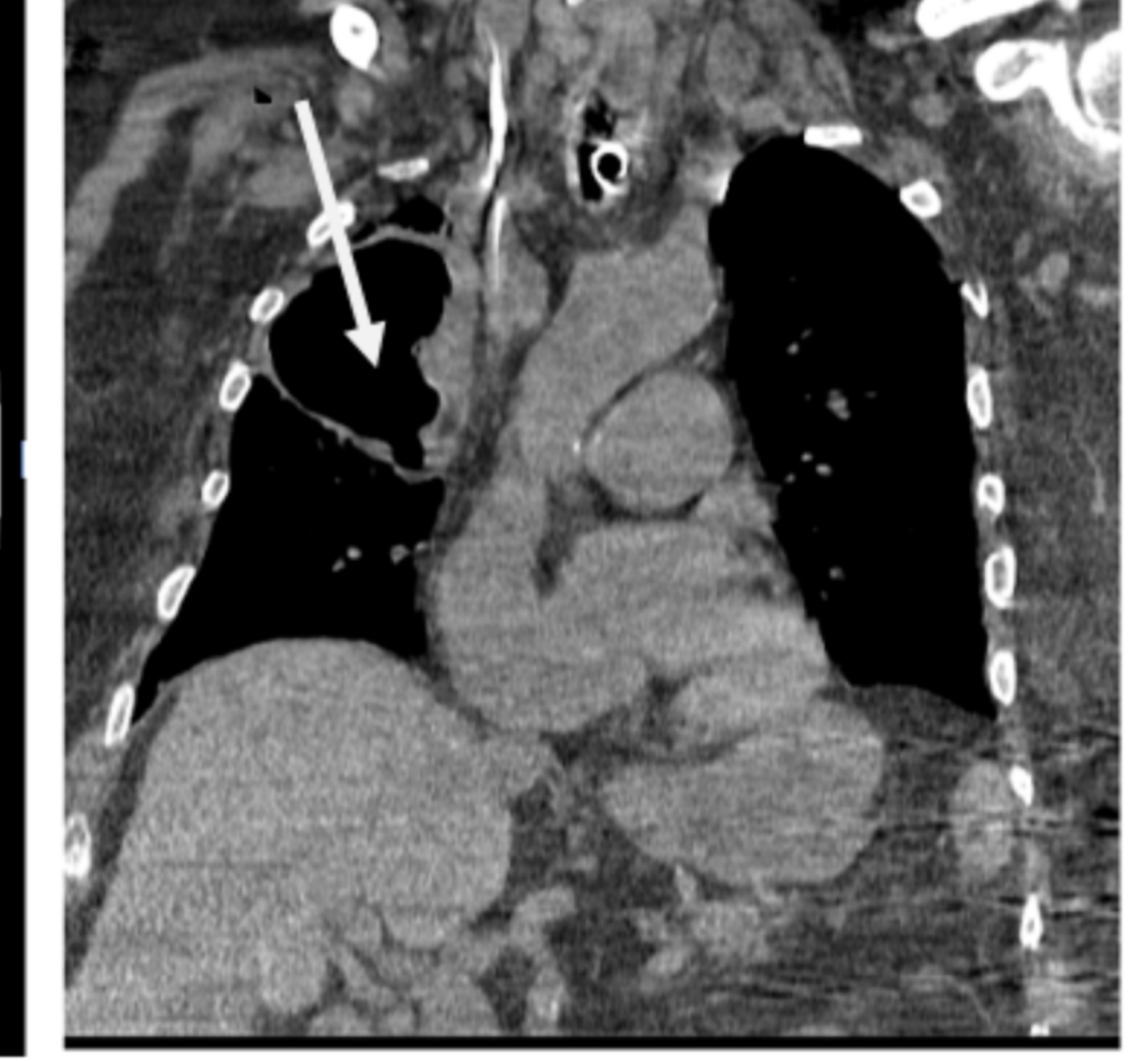
Chest CT scan: The pulmonary parenchyma demonstrates a large, thick-walled area of cavitation in the right upper lobe, measuring 7.3 x 5.4 x 7.5 cm. Multiple irregular nodules, quite small, less than 3 mm, peripheral to this area of cavitation in the right apex can also be seen.

There was patchy infiltrate versus atelectasis observed on the chest X-ray at the left lung base (Figure 1). The right upper lobe also had patchy infiltrates and a new thick-walled cystic airspace of 7 cm. A large, thick-walled cavitation area was demonstrated by the pulmonary parenchyma in the right upper lobe according to the chest CT scan, which measured 7.3 x 5.4 x 7.5 cm with less than 3 mm sized multiple irregular nodules that were peripheral to this cavitation area in the right apex (Figure 2). After admitting the patient to the medical intensive care unit (MICU), he was given broad-spectrum antibiotic while waiting for the culture results to treat sepsis that was possibly secondary to gram-negative/methicillin-resistant Staphylococcus aureus pneumonia. No growth was shown in urine and blood cultures. The patient’s respiratory status kept declining while awaiting results of the sputum culture and sensitivity, and heavy gram-negative bacillus was also observed in the preliminary respiratory culture. As a result, 500 mg of meropenem was given daily intravenously instead of cefepime.

The patient was started on polymyxin B three days later after the official sputum culture, and sensitivity report indicated the presence of A. faecalis. The bacteria were resistant to fluoroquinolones, Zosyn, cephalosporins, carbapenems, trimethoprim-sulfamethoxazole, and tobramycin. On the basis of multiple irregular nodules shown on the chest CT scan, three sputum cultures were sent, which returned negative for acid-fast bacilli. The patient was observed to be at a high risk of developing pneumothorax and as a result drainage of cavitary lesion through interventional radiology was deferred. The patient was given 600 mg of linezolid since the white cell count was increasing even though polymyxin B was being given. Repeat urine and blood cultures came out negative. In addition, the fungal culture was also negative. The repeat sputum culture showed A. faecalis. The patient’s condition worsened and he died despite the aggressive antibiotic treatment.

## Discussion

Currently, three species are included in the genus Alcaligenes namely Alcaligenes faecalis, Alcaligenes xylosoxidans, and Alcaligenes piechaudii. Previously, the species A. xylosoxidans was known as Achromobacter xylosoxidans. It can be divided on the basis of carbohydrate utilization studies into two subspecies called xylosoxidans and denitrificans [7]. The bacteria were discovered for the first time in feces, which led to it being named A. faecalis. It was found to be common in water, environments, and, later on, soil later. This microbe has an optimal temperature ranging between 20°C and 37°C. The R-mandelic acid is an important precursor essential to drug production of various kinds and can be produced by several strains of A. faecalis. This is why, nowadays, pharmaceutical industries are using the microbe extensively [8].

In most humans, the A. faecalis microbe does not cause systemic infections even though it exists in the alimentary canal. Humans with compromised immune systems as well as those with uncompromised immune systems have been observed suffering from infections caused by A. faecalis. Very few and rare cases have been reported that resulted in fatal outcomes such as appendicitis, abscesses, meningitis, bloodstream infection, endocarditis, and post-operative endophthalmitis [4,6]. In the literature, only one case can be found where A. faecalis was linked to lung abscess or pneumonia. Around the world, few hospitalized patients are suffering from infections caused by A. faecalis currently, and majority of them are in serious, life-threatening situations [9]. In the year 2004, the first bloodstream infection caused by A. faecalis was reported in immunocompromised cancer patients. It was found that the micro-organism was resistant to levofloxacin, monobactam, aminoglycosides, and ciprofloxacin [3]. A study was carried out on 188 patients, which revealed that 100% of A. faecalis isolates showed sensitivity to colistin, amikacin, and all cephalosporins; the ear discharge of 20 (10.6%) patients indicated the presence of A. faecalis; 75% of A. faecalis isolates showed sensitivity to sulfamethoxazole/trimethoprim; 90% of A. faecalis isolates showed sensitivity to tobramycin, tazobactam/piperacillin, and gentamicin; 82.6% of A. faecalis isolates showed resistance to levofloxacin; and 100% of A. faecalis isolates showed resistance to ciprofloxacin [10]. Complete resistance to all antibiotics available commercially was observed in the A. faecalis found in the sputum specimen of our patient. It was suspected that the bacteria were resistant to polymyxin B as well since the patient did not show any improvement even with the polymyxin B treatment.

## Conclusions

Serious illnesses and even death can be caused by A. faecalis, even though these organisms are considered to be an intestinal flora. Therefore, instead of contaminant, A. faecalis should be considered as pathogens because globally cases of life-threatening infections caused by A. faecalis are emerging, which are pan drug-resistant.
